# Role of microRNAs in doxorubicin-induced cardiotoxicity: an overview of preclinical models and cancer patients

**DOI:** 10.1007/s10741-017-9653-0

**Published:** 2017-09-25

**Authors:** Clarissa Ruggeri, Sonia Gioffré, Felice Achilli, Gualtiero I. Colombo, Yuri D’Alessandra

**Affiliations:** 1 Immunology and Functional Genomics Unit, Centro Cardiologico Monzino IRCCS, via Parea 4, 20138 Milan, Italy; 2 Department of Health Science, Cardiology Unit, San Gerardo Hospital, Monza, Italy

**Keywords:** Doxorubicin, microRNA, Cardiotoxicity, Biomarkers

## Abstract

Cardiotoxicity is a well-known side effect of doxorubicin (DOX), but the mechanisms leading to this phenomenon are still not completely clear. Prediction of drug-induced dysfunction onset is difficult and is still largely based on detection of cardiac troponin (cTn), a circulating marker of heart damage. In the last years, several investigations focused on the possible involvement of microRNAs (miRNAs) in DOX-induced toxicity in vitro, with contrasting results. Recently, several groups employed animal models to mimic patient’s condition, investigate the biological pathways perturbed by DOX, and identify diagnostic markers of cardiotoxicity. We reviewed the results from several studies investigating cardiac miRNAs expression in rodent models of DOX-treatment. We also discussed the data from two publications indicating the possible use of circulating miRNA as biomarkers of DOX-induced cardiotoxicity. Unfortunately, limited information was derived from these studies, as selection methods of candidate-miRNAs and heterogeneity in cardiotoxicity assessment greatly hampered the novelty and robustness of the findings. Nevertheless, at least one circulating miRNA, miR-1, showed a good potential as early biomarker of drug-mediated cardiac dysfunction onset. The use of animal models to investigate DOX-induced cardiotoxicity surely helps narrowing the gap between basic research and clinical practice. Despite this, several issues, including selection of relevant miRNAs and less-than-optimal assessment of cardiotoxicity, greatly limited the results obtained so far. Nonetheless, the association of patients-based studies with the use of preclinical models may be the key to address the many unanswered questions regarding the pathophysiology and early detection of cardiotoxicity.

## Introduction

Doxorubicin (DOX), one of the most renowned anti-cancer drugs, is widely used to treat several pediatric and adult tumors, including breast cancer, leukemia, and lymphomas. It belongs to the Anthracyclines family, whose first constituent, Daunorubicin, was isolated from *Streptomyces peucetius* in 1960s. One of the key features of these drugs is the ability to inhibit topoisomerase II, leading to double-stranded DNA breaks and thus hampering both cellular replication and transcription. Anthracyclines can directly intercalate into DNA, eventually leading to the disruption of physiological protein/DNA interactions (e.g., histones-DNA binding), and have been associated to the production of reactive oxygen species (ROS). As a result, DNA damage response pathways are perturbed in DOX-exposed cells and tissues [1–3].

Despite DOX-based therapies against cancer usually provide beneficial effects, leading to an increase in survival rates, drug-exposed cancer patients have a tangible risk of suffering from adverse cardiac effects (cardiotoxicity). These effects can range from subclinical ventricular dysfunction to severe cardiomyopathy, sometimes resulting in the need for heart transplantation [4]. Cardiotoxicity can occur either acutely or several years after treatment, and its occurrence is very difficult to predict, representing a major risk factor especially for childhood cancer survivors [5]. Since the discovery of the harmful effects of DOX on the heart, many researchers focused on investigating the mechanisms at the base of this phenomenon and on identifying reliable biomarkers for early diagnosis of DOX-induced toxicity. Regardless of the several hypotheses formulated through the years and of the results from decades of investigations, the whole picture is far from being understood. Indeed, several mechanisms of action at the base of drug-induced toxicity were proposed and experimentally corroborated (e.g., induction of oxidative stress and/or topoisomerase inhibition), but many remain unveiled. Increasing evidences indicate that DOX-induced cardiomyopathy is caused by the cumulative effects of several perturbed pathways, particularly the DNA damage response pathway, eventually leading to death and apoptosis of cardiac cells [6].

Patients either presenting or at risk of cardiotoxicity are usually treated with cardio-protective medications, such as angiotensin-converting enzyme (ACE) inhibitors, which help in mitigating the side effects of prolonged use of anthracyclines [4].

The assessment of cardiotoxicity onset can be achieved by means of several approaches: echocardiographic evaluation of cardiac function (usually by measuring left ventricular ejection fraction, LVEF), imaging, and endomyocardial biopsy [7, 8]. However, these techniques present important limitations, including low sensitivity, high invasiveness, elevated costs, and late detection of heart dysfunction.

Several investigations conducted on DOX-affected patients led to the identification of circulating, easily detectable markers of disease onset, thus addressing some of the issues of the abovementioned diagnostic techniques. Among others, brain natriuretic peptide (BNP) and cardiac troponins (cTn) demonstrated to be highly reliable and were adopted as robust circulating biomarkers of DOX-induced cardiotoxicity [9, 10]. In particular, in the clinical setting, increased levels of plasma BNP are associated with the presence of congestive heart failure (HF) [11]. Interestingly, congestive HF is characterized by impaired cardiac contractility and elevated wall stress, thus presenting many similarities with late-stage DOX-induced cardiac impairment. On the other hand, cTn are the gold standard markers of heart damage, being released in the circulatory system upon cardiac necrosis. This phenomenon characterizes, for instance, myocardial infarction and myocarditis [12, 13]. Nevertheless, since troponins levels increase in the blood only after tissue damage has occurred, they cannot be exploited as early diagnostic markers of dysfunction onset.

Considering the limitations of current biomarkers, there is an urgent need to identify new stable, highly sensitive, and noninvasive biomarkers. Recently, several studies turned their attention to the involvement of microRNAs (miRNAs) in DOX-induced toxicity. miRNAs represent attractive therapeutic targets in the cardiac context. This is due to the fact that these small (approximately 22 nucleotides), endogenous, single-stranded-RNAs regulate gene expression by either inhibiting messenger RNA (mRNA) translation or by promoting its degradation [14]. They play a key role in many biological processes, such as cell differentiation, replication, and regeneration, both under physiological and pathological conditions. Growing evidence has shown that miRNAs are involved in all cardiac functions, including conductance of electrical signals, heart muscle contraction, and growth. Anti-miRNA oligonucleotides, having complementary sequences to mature miRNA, have been used to determine miRNA functions and studied as potential miRNA-based drug targets [15]. Interestingly, miRNAs also represent noninvasive and specific circulating markers of several cardiovascular diseases [16–18].

Circulating miRNAs have many potential advantages as biomarkers: (i) they are evolutionarily conserved among species, allowing translation from preclinical models to clinical practice [19], (ii) some of them appear to be tissue-specific [20, 21], and (iii) they demonstrated to be highly stable over time in various body fluids, including plasma, serum [22, 23], urine [24], and saliva [25]. In addition, miRNA levels can be assessed by different sensitive techniques such as quantitative real-time PCR (RT-qPCR) [26] and next-generation sequencing [27].

**Fig. 1:**
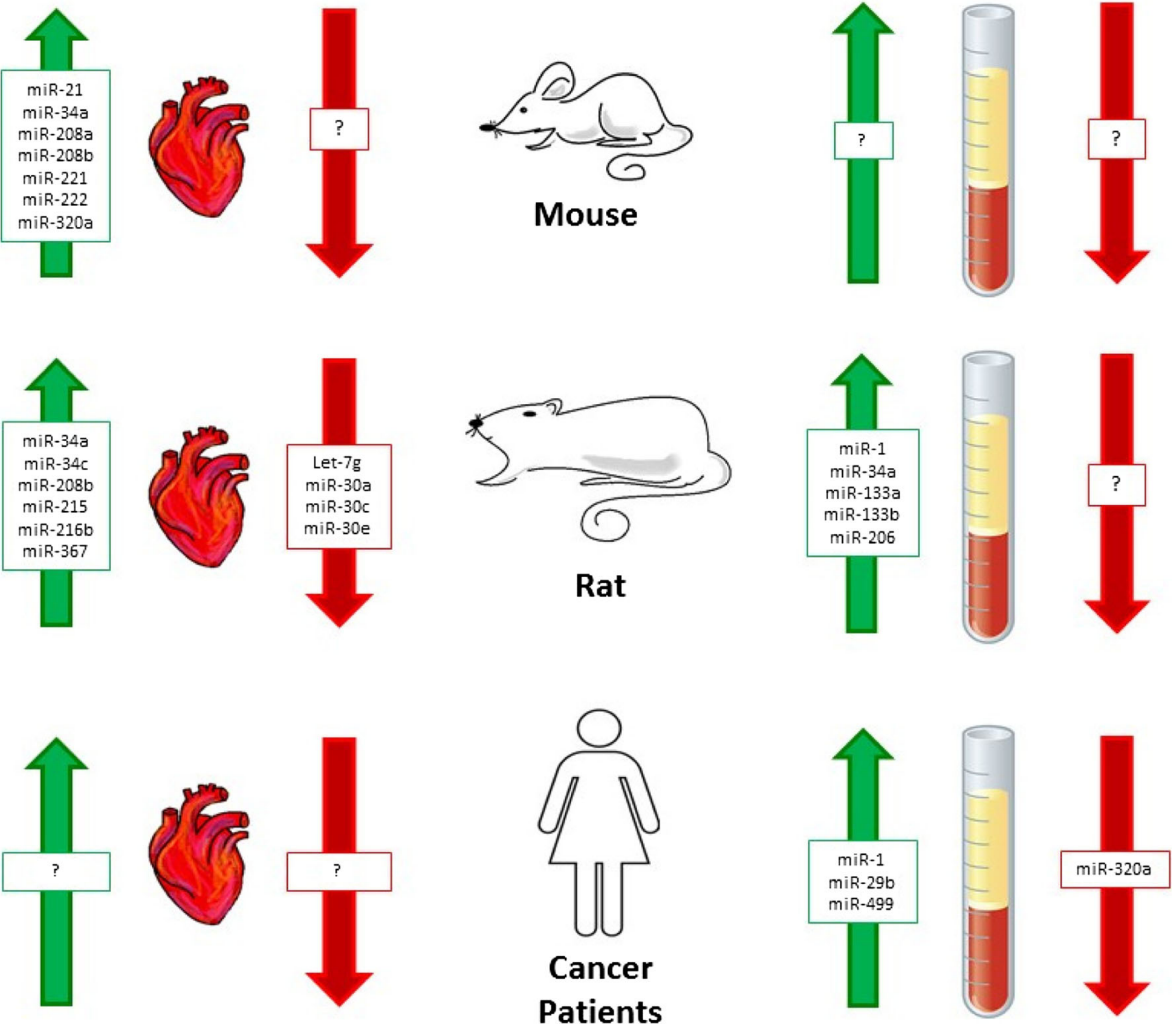
Summary of miRNA variations in hearts and plasma samples from all considered doxorubicin-treatment models and patients

Many research groups focused their attention on in vivo studies in order to try and confirm results from published in vitro studies concerning DOX and miRNAs, while at the same time attempting to overcome their limitations [28–30]. For obvious reasons, it is not possible to study the mechanism governing cardiotoxicity directly in cancer patients; thus, the clinical relevance of anthracycline-induced chronic cardiotoxicity has triggered many investigations based on the use of animal models, such as rats and mice, to shed some light on the several effects of DOX treatment. In vivo studies are important not only in the cardiac field but also, for example, to establish the teratogenic potential of this compound [31] and its harmful effects on the liver [32]. However, the prevalent focus of DOX-based preclinical studies is cardiotoxicity. Several investigations conducted through the years allowed the establishment of robust and reliable animal models, thus leading to important findings in terms of: unforeseen interactions of DOX with other drugs, discovery of cardioprotective molecules and treatments to contrast heart failure onset and progression, assessment of new formulations, and protocols for drug delivery [33, 34]. Here, we present an overview of the past and recent studies conducted to investigate the role of miRNAs in DOX-mediated cardiac dysfunction, discussing their strengths and limitations. All presented works are summarized in Table 1 and results are depicted in fig. 1. For clarity’s sake, we decided to discuss separately tissue-based studies from those investigating circulating miRNAs.

**Table 1:** Overview of the past and recent studies conducted to investigate the role of miRNAs in DOX-mediated cardiac dysfunction.

PMID	Model	Sample	miRNA selection	Regulated miRNAs	Cardiotoxicity assessment
20495188	Mice	Cardiomyocytes	Literature	miR-146a	No evaluation
22301161	Rats	Heart	Literature	let-7 g	Variation in: body weight, heart rate, pulse pressure, and heart organ coefficient. Cardiac troponin T.
22859947	Rats	Heart	Screening	miR-34c, miR-208b, miR-215, miR-216b, miR-367	Macro- and micro-vacuolation of cardiac myocytes
25448438	Mice	Heart ventricles	Screening	miR-21, miR-34a, miR-208b, miR-221, miR-222	Cardiac troponin T. Histological assessment of cardiac cell damage.
26132560	Mice	Heart	Literature	miR-21	Cardiac imaging,heart indices (heart weight/body weight ratio)
25092230	Rats	Plasma	Literature	miR-1, miR-133a, miR-133b, miR-206	Cardiac troponin T and I, skeletal TnI concentration
25950484	Rats	Cardiomyocytes from treated animals	Literature	miR-30 family	Cardiac imaging
26137188	Mice	Heart	Literature	miR-208a	Cardiac imaging
27633839	Rats	Heart	Literature	miR-30a, miR-30c, miR-30e	Cardiac imaging
26837315	Mice + cancer patients	Heart (mice); blood (patients)	Literature	miR-320a	Cardiac imaging
27694688	Rats	Heart; plasma	Literature	miR-34a	Cardiac imaging
26046768	Cancer patients	Plasma	Literature	N/A	Variation in cardiac troponin I (cTnI) and left ventricle ejection fraction (LVEF)
28052002	Cancer Patients	Plasma	Literature	miR-1	Cardiac imaging and cardiac troponin I (cTnI)
28377429	Cancer patients	Plasma	Literature followed by screening	miR-1, miR-29b, miR-499	Cardiac imaging and high sensitivity cardiac troponin T (cTnT)

## Tissue-based studies

### Doxorubicin regulation of ErbB4 via miR-146a

In 2010, Horie and colleagues were the first to publish a manuscript suggesting a perturbation of miRNA expression in an animal model of DOX-induced cardiotoxicity [35]. The authors described a significant reduction in ErbB2 Receptor Tyrosine Kinase 4 (ErbB4) expression in the heart of maleC57BL/6 mice, 1 day after treatment with 20 mg/g DOX. Since the proteasome was only partially involved in the drug-induced downregulation of ErbB4, miRNAs were hypothesized as possible mediators of the drug effects. In particular, miR-146a was selected as a candidate because of its abundant expression in the heart and because it potentially targets *Erbb4* mRNA. Indeed, In vitro experiments on neonatal rat cardiomyocytes (NRCM) showed a decrease in ErbB4 protein expression and a concomitant increase of miR-146a levels upon treatment with 1 μM DOX for 24 h. Interestingly, overexpression of miR-146a in the same cells led to a decrease of ErbB4 protein levels but not of its transcript. Further, luciferase experiments confirmed *Erbb4* 3′UTR as a bonafide target of miR-146a. These results, along with the observation that concomitant DOX treatment and miR-146a overexpression increased cell death and that this was reverted by ectopic ErbB4 expression, led the authors to hypothesize that DOX-induced apoptosis could be the result of miR-146a mediated down-regulation of ErbB4. Despite the massive extent of in vitro results, the existence of a mechanism of induction of miR-146a in vivo as substantial causal mechanism of DOX-induced cardiac toxicity was not supported by any experimental data. Surprisingly, the authors did not evaluate miR-146a expression in the animal model, thus any claim about a physiological role of the miRNA in DOX-induced cardiotoxicity onset should be regarded as a speculation. Another potential issue about this work, is the amount of drug administered to the mice. Remarkably, the authors indicated in their manuscript that drug-treated animals received 20 mg/g of doxorubicin, an amount that would be startlingly high in comparison both with the medium dose usually administered in this kind of experiments (18–20 mg/kg). Careful evaluation of the manuscript indicated as reference for animal treatment revealed that the correct quantity of drug administered to the animals was 20 mg/kg.

### Cardiac perturbation of let-7g in DOX-treated rat hearts

In 2012, Fu and colleagues published a work investigating the possible effects of DOX-treatment on cardiac let-7g miRNA expression in vivo [36], although the exact reason behind the choice of let-7 g as the focus of their investigation is not clear. The authors used 20 adult male Wistar albino rats, divided into four groups, and intraperitoneally administered either saline or 3 mg/kg of DOX every other day up to the cumulative doses of 6, 12, and 18 mg/kg. Notably, only one control group was established and treated similarly to the DOX 18 mg/kg cohort. All rats were sacrificed 24 h after last injection, when predefined cumulative dose amount was reached. A few general indicators of heart function were measured to assess cardiotoxicity onset: body weight, heart rate, pulse pressure, plasma cardiac troponin T (cTnT) concentration, and heart organ coefficient (not clearly defined). The only marker really related to cardiac damage, cTnT, showed a slight but significant increase only in the 18 mg/kg group (vs. control animals). Of note, the investigators showed values for body weight and other parameters at day 12 for the 6 and 12 mg/kg DOX groups, but these groups were described as reaching their target cumulative dose at the 2nd and 4th administration (day 4 and 8, respectively), being then sacrificed 24 h later. Strangely, let-7g expression data in the heart was reported as performed in eight rats/group, while only five animals for each group were indicated in the methods section. In addition, there is no clear and exhaustive description about how and why U6 small nuclear RNA (snRNA) was adopted for normalization, beside the statement that “it appeared to be present in all cells”. In vivo, let-7g presented a significant downregulation only in the 18 mg/kg DOX-treated mice group, together with a decrease in heart rate. The expression of the miRNA declined also after acute (24 h) drug administration (at concentrations ranging from 0.004 to 0.5 μmol/l) in primary cardiac cells isolated from 1-day-old Wistar albino rats. Cells viability and visually registered beat frequency were negatively affected as well. The observed discrepancy in terms of time of dysfunction onset between in vivo and in vitro experiments could be due to a systemic response to the drug-induced damage in the animals, which is obviously lacking in cultured cells.

In addition, the authors speculated a role for the miRNA in DOX-induced cardiotoxicity, claiming that plasma concentration of let-7g in DOX-induced heart injury model showed a good correlation with cTnT and other indicators of cardiac function. Unfortunately, these data were not shown in any part of the manuscript. Remarkably, the authors focused their discussion mostly, if not completely, on data from the literature about possible targets of let-7g, conceivably involved in the heart impairing effects of the drug. No attempt to experimentally verify their speculations was presented. In conclusion, the contribution of this work to the understanding of the mechanisms of DOX-induced cardiotoxicity occurrence is not clearly appreciable.

### miRNA screening in DOX-treated rats

The same year, Vacchi-Suzzi and colleagues [37] reported that chronic myocardial toxicity induced by DOX in rats was associated with the modulation of several miRNAs and transcripts of several cardiomyopathy-related genes. In this study, six male rats/group were injected once a week respectively with DOX 1–2–3 mg/kg, dexrazoxane (DZR, a cardioprotective agent) 50 mg/kg, a combination of DOX 2 mg/kg and DZR, etoposide (EPS, a topoisomerase II inhibitor devoid of cardiovascular toxicity) 1–3 mg/kg, or saline for 2, 4, or 6 weeks. Thus, the cumulative dosage of DOX ranged from 2 to 18 mg/kg. Notably, only 2/6 animals survived 6 weeks dosing of DOX 3 mg/kg/week, possibly because of drug-induced systemic damage, although the causes of death were not indicated. As indicator of cardiotoxicity, the authors reported that DOX induced macro- and micro-vacuolation in cardiac myocytes, in both atria and ventricles, but the fraction undergoing vacuolation was small. Administration of 3 mg/kg/week of DOX induced time-dependent modulation in the expression of cardiac mRNAs previously associated with molecular responses to cardiomyopathies. Following heart damage onset, the authors conducted a miRNA screening on rats treated with 6 and 12 mg/kg DOX cumulative doses. After profiling of 518 rodent miRNAs using TaqMan low-density arrays qPCR platform, they detected approximately 370 microRNAs in all samples, arbitrarily choosing U6 snRNA as internal normalizer. When compared to control animals, the 6 mg/kg DOX group showed no appreciable perturbation in miRNA expression, while the 12 mg/kg cumulative dose led to increased levels of 17 miRNAs and to a decrease of 8 miRNAs. Among these, a subset of 10 miRNAs with the lowest nominal *p* values was selected for validation using single TaqMan assays performed on animals from the four groups treated with 4/6/8/12 mg/kg DOX. Five miRNAs (miR-34c, miR-208b, miR-215, miR-216b, and miR-367) displaying a consistent response to DOX were chosen for further expression profiling across all study groups.

In the case of animals treated with DOX alone, the authors observed a slight upregulation of miR-208b in the heart of all animals treated with mid- (2 mg/kg) and high- (3 mg/kg) dose of DOX for 2 weeks. At 4 weeks, the levels of all five miRNAs were significantly increased and dose dependency was observed. After 6 weeks, the levels of miR-208b, miR-215, miR-216b, and miR-367 were significantly increased, but dose dependency was difficult to assess since only two animals survived in the high-dose group. DZR treatment did not affect the physiological expression of investigated miRNAs. Interestingly, the cardioprotective agent was not able to contrast the DOX-induced miRNA upregulation, despite being able (as suggested, but not shown by the authors) to lessen the magnitude of the DOX-induced vacuolation. Among the EPS-administered animals, only minimal perturbations were observed regardless of dose and time. Taken together, these data suggested a DOX-specific regulation for all investigated microRNAs. Interestingly, the expression of three of the five miRNAs showed a positive correlation with the severity of cardiac tissue lesions. Indeed, increasing doses of DOX led to higher vacuolation grading in parallel with a progressively stronger upregulation of miR-208b, miR-216b, and miR-367. Of note, although low-dose treatment did not cause any detectable vacuolation, the authors observed a significant increase of miR-216b in this group. These results suggest that DOX-induced perturbations of a few specific cardiac miRNAs can precede (and possibly trigger) histopathological lesions of cardiotoxicity.

A potential major point of interest of this work relies in the genome-wide gene expression profiling conducted along with the miRNA screening, potentially allowing the authors to investigate miRNA/target interactions with functional relevance in cardiac response to the drug. Surprisingly, bioinformatics prediction showed no findings worth reporting. In conclusion, the predictive potential of a cluster of miRNAs as early tissue indicators of drug-induced cardiac lesions represent the major finding of this work. Remarkably though, no real direct evaluation of cardiac function was conducted beside the histological assessment of cellular damage.

### miRNA screening in DOX-treated mice

Identifying biomarkers of early DOX-induced cardiac tissue damage was also the scope of Desai and collaborators in a paper published in 2014 [38]. They investigated the expression of miRNAs in the hearts of male B6C3F_1_ mice chronically treated by injecting intravenously 3 mg/kg DOX (or saline) once a week for 2, 3, 4, 6, and 8 weeks, resulting respectively in cumulative DOX doses of 6, 9, 12, 18, and 24 mg/kg. Interestingly, the authors chose to sacrifice animals a week after each cumulative DOX exposure. The presence of cardiac injury was determined basing on cTnT plasma levels, significantly increased only in mice exposed to cumulative DOX doses of 18 and 24 mg/kg. Of note, mouse cTnT plasma concentrations were measured using the Elecsys Troponin T STAT assay by Roche Diagnostics, which is designed and routinely adopted to detect cTnT of human origin, but no data about validation and reliability in a rodent model were provided. Histologic assessment of cardiac cell damage was also conducted by light microscopy. Remarkably, only the 24 mg/kg DOX group showed signs of cardiac lesions, albeit their severity was minimal. Unfortunately, no imaging assessment of cardiac function was performed.

Despite the lack of evidence of heart lesions in mice exposed to DOX below 18 mg/kg, the authors used Agilent mouse miRNA microarrays v2 to investigate the expression of 1179 unique miRNAs in a total of 40 samples (“2 treatments × 5 cumulative exposures × 4 samples per group”). Two hearts from mice in each treatment group with comparable cTnT plasma concentrations were pooled and used for total RNA extraction. Interestingly, only miR-34a expression showed a significant increase at all DOX doses, and this was confirmed by single RT-qPCR assay and indicated as an early molecular marker of cardiac tissue injury during DOX treatment. Of note, drug exposures to 18 and 24 mg/kg influenced the expression of 8 and 21 miRNAs, beside miR-34a. Hypertrophy-related miRNAs (miR-21, -221, -222, and-208b) were increased at both doses. The cardiac upregulation of these four miRNAs, together with the rise of plasma cTnT concentration at both 18 and 24 mg/kg DOX, might suggest their association with cardiac damage. Unfortunately, the lack of assessment of cardiac function does not allow any strong conclusion about this hypothesis.

### Role of miR-21 in acute and chronic DOX treatment

In 2015, Tong and colleagues investigated the behavior of a single specific miRNA, miR-21, in response to DOX-triggered cardiotoxicity in a mouse model [39]. This choice was driven by the known activation of this miRNA in cardiovascular diseases, including myocardial infarction and cardiac fibrosis [40, 41], and by its anti-apoptotic function in ischemia/reperfusion- and hypoxia/re-oxygenation-induced cardiomyocyte apoptosis [42]. The authors used wild-type Balb/c male mice to mimic both acute and chronic DOX administration. In the acute setting (A-DOX), mice were continuously injected intraperitoneally with either saline or DOX for 5 days with a cumulative dose of 20 mg/kg. In the chronic setting (C-DOX), mice were injected with DOX 5 mg/kg/week for 4 weeks, again with a cumulative dose of 20 mg/kg. The animals were sacrificed 1 day after reaching the cumulative dose following assessment of cardiotoxicity by cardiac hemodynamic measurements. Both DOX treatments resulted in a diminished survival rate, depressed cardiac function, and decreased heart indices (heart weight/body weight ratio), with cardiac injury being more pronounced in the C-DOX group as confirmed by apoptosis evaluation with TUNEL assays. Assessment of miR-21 expression levels was performed both in vivo and in vitro by single RT-qPCR assay, arbitrarily using U6B snRNA (RNU6B) as internal reference. Notably, miR-21 showed a modest increase (1.8-fold vs. controls) in the myocardium of chronically treated animals, while no perturbation was found in acutely treated animals. Moreover, the rate of apoptosis was very high in the C-DOX group, while it was less increased in the A-DOX group (31.67 vs. 9.01% respectively, in comparison with controls for which a value was not indicated but appeared to be around 2%).

Following in vitro experiments using DOX-treated (0–4 μM for 24 h) heart tissue-derived H9C2 cells, a dose-dependent increase in miR-21 expression levels was detected. Transfection of these cells with miR-21 mimic molecules induced a mild increase in the expression of the miRNA, while transfection with synthetic inhibitors led to a 0.55-fold reduction. A 18–37% decrease of apoptosis was observed when miR-21 was overexpressed in DOX-treated cells, while an increase of up to 57% was registered using the inhibitors. Curiously, the authors did not show the effects of miR-21 modulation in the absence of DOX. Following bioinformatics prediction of miR-21 targets, experiments were performed to investigate expression of B cell translocation gene 2 (Btg2), a key gene involved in cell differentiation, proliferation, DNA damage repair, and apoptosis in cancer cells [43]. *Btg2* expression decreased both in myocardium of DOX-administered animals and in H9C2 cells treated with the drug. As a take-home message, the authors indicated miR-21 as a cardiomyocyte protector, suggesting that its regulation of Btg2 might contrast DOX-induced injury. However, despite the validation of Btg2 as a bonafide target of miR-21, its role in apoptosis remains unclear. In addition, the parallel strong increase of apoptotic rate and miR-21 expression in vivo seems to be in contrast both with the known anti-apoptotic activity of the miRNA and with the in vitro results.

### Perturbation of miR-30 family by cardiac insults

One of the most articulated investigation in terms of experimental design was published in the same year by Roca-Alonso and coworkers [44]. In this case, the authors chose to take advantage of the combined results from three different models of cardiotoxicity in order to identify a robust heart-damage response miRNA signature. In particular, two models of cardiac injury were compared in male Sprague–Dawley rats: proximal left anterior descending (LAD) coronary artery ligation, inducing myocardial infarction (MI), and DOX administration. MI animals were sacrificed 4 weeks after surgery and hearts were explanted. In the DOX-treatment model, rats were administered with a cumulative dose (15 mg/kg) of DOX, delivered via six intraperitoneal injections over 2 weeks, and sacrificed 3 weeks after the last injection. Animals were constantly monitored for signs of HF. Indeed, dilated cardiomyopathy occurrence was demonstrated by reduction of left ventricular (LV) systolic function at sacrifice. In addition, DOX-treated hearts showed increased LV end-systolic and end-diastolic volumes and reduced contraction efficiency using pressure-volume analysis, consistent with a dilated cardiomyopathy phenotype. Remarkably, no cardiac functional assessment was shown for the MI group. After sacrifice, ventricles were separated from the atria and used to obtain cardiac cells for both MI and DOX rats. Cardiomyocytes were isolated basing on “centrifugation speed and size,” and viable cells selected by plating on laminin-coated plates before further use. The third model consisted of rodent ventricular cardiomyocytes derived from control rats and treated in vitro for 6 h with DOX 1 μmol/l, a dose that the authors stated to be “clinically relevant.” Regrettably, the authors gave no indication about the method used to establish the clinical relevance of the drug concentration, the number of treated cells, or the method used for RNA extraction.

They performed a miRNA profiling using the nCounter Rat miRNA Expression Assay on the three different models. Evaluation of the results obtained comparing “treated vs. control” evidenced seven miRNAs with perturbed expression in all groups: miR-29c, miR-30d, miR-30e, miR-133b, miR-143, miR-210, and miR-345-5p. Remarkably, all of them showed reduced expression in comparison with controls. The authors selected miR-30e, miR-210, and miR-29c for validation by RT-qPCR in vitro, and snRNA U6 was arbitrarily chosen as a normalizer. A very good correlation was observed between profiling and validation results. Since three members of the miR-30 family (miR-30a, miR-30d, and miR-30e) were downregulated in at least two models, the authors focused their attention on them. Pathway enrichment analysis of putative miR-30 targets revealed a potential implication in cardiomyopathy. Indeed, miR-30 expression seemed to attenuate the contractile response of cardiomyocytes to β-adrenoceptor (βAR) stimulation. Similarly, Bax/Bcl-2 ratio (an indicator of the susceptibility of a cell to apoptosis) showed a decrease in DOX-treated cardiac cultured cells upon miR-30e overexpression, indicating a beneficial effect of miR-30 expression for cardiac cell viability [45]. To support the importance of miR-30 family in the DOX-response, the authors proposed as mechanism of regulation the inhibition of miR-30 cluster transcription by GATA6 (a transcription factor known to play a key role in cardiac development). Indeed, they showed that DOX treatment could induce GATA6 expression, thus triggering miR-30 downregulation. These results led the authors to conclude that miR-30 family are potential cardioprotective molecules against anthracycline-induced cardiotoxicity.

This study was the first to address the potential involvement of miR-30 family in response to DOX possibly opening the door to future therapeutic applications. Unfortunately, as stated by the authors, the experiments to address this issue could not be done due to the restrictions applied on animal experimentation in UK. Thus, despite the use of animal models, this investigation should be considered an in vitro study. It is not entirely clear why the authors decided to isolate cardiomyocytes instead of investigating miRNA levels in whole heart tissues. Another consideration should be made about the choice of in vivo models. LAD ligation induces tissue necrosis in the LV, while DOX-induced injury is characterized by systemic damage of all tissues, including all cardiac districts. Since cardiomyocyte isolation was conducted using both ventricles, the unbalance between cells originating from either RV or LV could have impacted the final results. Interestingly, an almost concomitant work from Shen and colleagues [46] suggested that inhibition of miR-30 family expression was protective against cardiac ischemic injury in a mouse model. These contrasting data cast some doubts about the real role of these miRNAs in heart damage.

### miR-208a: perturbation and possible therapeutic application in response to DOX

In a later study, Tony and coworkers investigated miR-208a potential in contrasting DOX cardiotoxicity because of its known heart specificity and its role in response against cardiac stress [47]. They used female Balb/C mice in three different settings: sham-treated, DOX-treated, and DOX-plus-antagomir-treated animals. Sham and DOX-treated mice were injected with saline in tail vein 4 days before being administered either with phosphate buffered solution (PBS) or with a single intraperitoneal dose of DOX (20 mg/kg), respectively. Similarly, antagomir mice were administered with 50 nmol of miR-208a-antagomir 4 days before being treated with DOX (20 mg/kg). After 7-day follow-up, mice were sacrificed and hearts explanted. Regrettably, no scrambled antagomir was used as control for silencing effects. Of note, a high lethality rate (47.8%) was observed in DOX mice, which was reduced to 12.5% in miR-208a antagomir-treated animals at 7 days. The authors observed a moderate upregulation (fourfold) of miR-208a in DOX heart tissues in comparison to sham mice, while the miR-208a-antagomir group showed no regulation by the drug. Remarkably, the authors gave no indication about how normalization of miR-208a expression levels was performed. GATA4, a transcription factor involved in cardiac development and hypertrophy [48] known to be a target of miR-208a [49], was negatively regulated in the DOX group, while the antagomir reverted this effect. Interestingly, previous studies showed that DOX-induced cardiomyocyte apoptosis is, at least in part, mediated by GATA4 [50], suggesting a role for miR-208a in this pathway and a possible exploitation of this miRNA to avert the toxic effects of the drug on cardiomyocytes. In order to prove this point, they measured *Bcl-2* (an anti-apoptotic gene) expression levels and apoptosis rate in the heart samples from DOX and antagomir-treated mice (but not from sham mice). *Bcl-2* showed a slight increase in the antagomir group. Consistently, the authors observed augmented apoptosis rates in DOX mice hearts, which appeared to be counteracted by blockage of miR-208a. Beside these molecular effects, treating mice with a specific antagomir seemed to have functional implications, preventing cardiac dysfunction. In conclusion, the authors suggested that inhibition of miR-208a could have beneficial applications in attenuating DOX-induced myocyte apoptosis. The use of systemically administered antimiRs in exploring miRNA function in rodents and primates has been previously proposed [51], thus the article by Tony and coworkers supports the potential of these compounds as a new class of therapeutics for disease-associated miRNAs.

### DOX regulation of miR-320a in heart tissue and blood

A recent study from Yin and colleagues investigated miR-320a expression in induced cardiotoxicity both in vivo and in a limited group of human plasma samples [52]. In this work, male C57BL/6 mice were acutely treated with DOX (single intraperitoneal injection, 25 mg/kg) or saline for 14 days, before organ collection. This group previously reported that miR-320a contributes to atherogenesis [53]. Starting from this observation, they investigated miR-320a possible regulation by DOX, even if the rationale is not clear. In the manuscript, they described a slight upregulation of miR-320a levels (arbitrarily normalized on U6 snRNA expression) in hearts and lungs, but not in kidneys and livers, of DOX-treated animals. Similarly, they observed a comparable regulation in H9c2 and HUVEC cells treated with DOX 5 μM for 12 h.

Basing on these results, the authors focused on the possible mechanistic role of miR-320a in DOX-induced cardiotoxicity, using recombinant adeno-associated virus (rAAV) to modulate its expression in vivo. In particular, miRNA upregulation was obtained using a rAAV vector containing a miR-320a expression cassette, while its inhibition was obtained by the “tough decoy RNA” (TuD) technology [54]. In total, five treatment groups were investigated: DOX alone (CTRL), DOX + rAAV expressing GFP (GFP-rAAV), DOX + miR-320a rAAV (rAAV-320a), DOX + rAAV inhibiting miR-320a (rAAV-320a-TuDs), DOX + rAAV expressing a mutated miR-320a (rAAV-320amut). Evaluation of miR-320a expression showed a DOX-mediated upregulation (fourfold vs controls), which was additionally increased by treatment with rAAV-320a (sixfold vs controls). MiRNA inhibition led to a 50% reduction vs. CTRL and GFP-rAAV. Echocardiography showed a detrimental effect of 320a overexpression on heart function, while a slight improvement was observed in mice treated with 320a inhibitors. Normal heart functions, though, were very far from being recovered. Noteworthy, the effect of miR-320a modulation in vivo without concomitant DOX treatment was not investigated. Interestingly, the drug induced the upregulation of cardiac mRNA levels of BNP, which is secreted by ventricles in response to excessive stretching and is increased in several cardiovascular pathologies [55]. This increase was contrasted by inhibition of miR-320a, showing its mild ability to attenuate cardiac impairment due to the drug. Since endothelial cell injury is a key event of vascular injury, the authors investigated the possible role of miR-320a in this phenomenon by treating HUVEC with DOX. Indeed, the drug induced an impairment of endothelial cell migration and tube formation. Moreover, miR-320a inhibition relieved DOX-induced inhibition of cell proliferation and promotion of apoptosis, while miR-320a overexpression aggravated these effects. Similarly, HUVEC transfected with miR-320a exhibited impaired nitric oxide (NO) release, tube formation, and cell migration. After target prediction, the authors validated VEGF as a target of miR-320a in vitro, implying that the VEGF pathway could be a possible mediator of miR-320a response to DOX. Nonetheless, since miR-320a led neither to beneficial effects on mortality rates nor to any functional recovery, a clinical application of this miRNA as a therapeutic agent against DOX cardiotoxicity seems very unlikely. Of note, mir-320a expression was measured, for the first time, also in the blood of patients with acute myelogenous leukemia (AML) treated with anthracycline-combined chemotherapy. Circulating miR-320a levels was found downregulated in five DOX-AML subjects compared to five control donors, but no information was given about the type of sample analyzed (plasma or whole blood), timing of sample collection and processing and, more importantly, about the assessment of cardiotoxicity onset in any patient. In addition, the comparison with healthy donors is questionable for treatments that imply several cycles of drug administration; in such cases, the baseline levels of miRNA expression should be assessed before the therapy and used as reference.

### Dox-induced autophagy is mediated by miR-30

A major cause of drug-induced cardiomyopathy is the prolonged activation of myocardial autophagy. Generally, autophagy is a cell-regulated mechanism that allows turnover of cellular components, and dysregulation of this balance can result in tissue damage [56]. Recently, miR-30 emerged as a key player in autophagy [57], thus Lai and coworkers focused their attention on its involvement in the cardiac-cell autophagy process [58]. In addition, since several studies pointed out a beneficial action of ACE2 on cardiac function, such as reduction of angiotensin II-mediated cardiac hypertrophy and fibrosis [59], they investigated its possible role in autophagy. Indeed, although no direct evidence links ACE2 to this process, overexpression of ACE2 has been observed in murine autophagy-deficient embryonic fibroblasts [60]. Male Sprague-Dawley rats were divided into three groups: control untreated rats, DOX-treated rats, and recombinant human ACE2 (rhACE2)-administered DOX-treated rats. Regrettably, the number of animals used for each treatment was not indicated. Cardiomyopathy was induced by intraperitoneal administration of six doses of the drug for 2 weeks (15 mg/kg cumulative dose). In addition, rhACE2 2 mg/kg was given for 4 weeks (8 mg/kg total dose) after DOX administration. Four weeks after treatment, DOX rats developed a progressive decrease in cardiac function measured by echocardiography (compared with controls), and a mortality rate of 32% was observed. On the contrary, ACE2 administration greatly improved all cardiac parameters, which were almost undistinguishable from those of untreated animals. Evaluation of miR-30 family (miR-30a, miR-30c, and miR-30e) expression normalized on U6 snRNA in heart tissues from all rats showed a marked decrease for these miRNAs in DOX-administered animals. Of note, treatment with rhACE2 increased miR-30a and miR-30e expression. These results are in keeping with previously published results [44], reporting miR-30 downregulation in adult rat ventricular cardiomyocytes derived from DOX-treated rats. Interestingly, animals treated with both rhACE2 and DOX showed reduced autophagy and apoptosis in comparison to DOX animals, as indicated by levels of Beclin-1 and LC3 cytosolic form II over I ratio and caspase 3 activity, respectively [61, 62]. In addition, since Beclin-1 is a target of miR-30e [63], the authors modulated miR-30e expression in primary cardiomyocytes. They found that miR-30e overexpression attenuated DOX-induced cell apoptosis, while its inhibition reversed heart-protective effect of ACE2. These results hint a possible therapeutic application of autophagy regulation, contrasting the detrimental effects of DOX by ACE2 treatment and, possibly, via miR-30e.

### miR-34a involvement in cardiotoxicity: player, biomarker, or both?

Among known miRNAs involved in cardiac function, miR-34 family members participate in cell cycle, senescence, apoptosis, differentiation, and in heart response to stress [64]. In particular, earlier studies showed the therapeutic effect of miR-34a inhibition as potential cardioprotective intervention [15]. Moreover, miR-34a was indicated as possible plasma biomarker of HF onset following acute myocardial infarction [18]. In their recent investigation, Piegari and colleagues explored DOX effects on miR-34a expression in rat cardiac progenitor cells (rCPCs), studying both its predictive capacity as a circulating biomarker of cardiac damage and its possible role in protecting the heart from chemotherapy-related toxicity [65]. Firstly, miR-34a expression (arbitrarily normalized using snRNA U6) was evaluated upon treatment with DOX 0.5 μM for 24 h in rCPCs and cardiac fibroblasts (CFs) isolated from female Fisher 344 rat hearts, in rat embryonic myoblasts (H9c2 cells), and in rat aortic endothelial cells (RAOEC). Increased miR-34a was found in both rCPCs and RAOEC and in their culture media, in CFs (cells only), but not in H9c2. Then, since their previous work indicated CPC loss as a cause of anthracycline toxicity [66], the authors evaluated whether miR-34a block could contrast this phenomenon. Indeed, its inhibition 24 h before DOX administration reduced the cytotoxic effects of the drug, significantly increasing rCPCs vitality and proliferation. Importantly, the same treatment did not affect DOX cytotoxic activity on MCF7 breast cancer cells, even when anti-miRNA was used at a concentration up to 10 times higher concentration than on rCPCs. Previous works had shown that miR-34a can trigger apoptosis in tumor cells [67] and cardiomyocytes [68]. In addition, the authors investigated whether miR-34a could affect also cell death. Indeed, TUNEL assay showed a marked reduction of apoptotic cells after miR-34a inhibition in DOX-rCPCs. Of note, the expression of a known miR-34a target, Bcl-2 [69], evaluated both by RT-qPCR and Western blot, was significantly increased after anti-miRNA administration both at mRNA and protein level. Remarkably, miR-34a inhibition resulted also in SIRT1 upregulation, thus protecting rCPCs from DOX-induced damage and cell senescence. SIRT1, in fact, is a solid miR-34a target regulating cell cycle, survival, and senescence [70].

One of the major novelty of this work was the attempt to measure the paracrine effect of miR-34a secreted by rCPCs on other cells. Indeed, the authors used conditioned media from DOX-treated rCPCs (with or without miR-34a inhibition) to culture H9c2 cells, CFs, and RAOEC for 48 h. They observed a reduction in apoptosis and senescence in H9c2 and RAOEC cells, but not in CFs. Curiously, only cells that usually adopted different growth media from that of rCPCs were affected by the conditioned medium. The authors indicated miR-34a as the only plausible paracrine effector, without discussing the possibility of other molecules secreted by the cells upon treatment. In addition, they suggested that pharmacological inhibition of miR-34a could revert these effects, but did not present any experimental evidence.

Using a previously established animal model of DOX-induced cardiotoxicity [71], the authors investigated whether the drug could trigger miR-34a secretion from the heart and other tissues. In particular, F344 rats were administered a cumulative dose of DOX 15 mg/kg by six intraperitoneal injections over a period of 2 weeks. Treated and control animals were sacrificed 3 weeks after the first injection, and cardiac function was assessed by echocardiography. DOX animals showed LV dysfunction (10–20% decrease in LVEF and fractional shortening). Evaluation of miR-34a expression (normalized to snRNA U6) indicated a DOX-induced upregulation in the heart, liver, kidney, and skeletal muscle. miR-34a baseline levels were higher in cardiac tissues than in the other organs, but the DOX-triggered increase was modest in the heart and more pronounced in both liver and skeletal muscle. Further, in situ hybridization in heart sections of DOX-treated rats confirmed the increase of miR-34a in rCPCs identified as c-kit-positive cells. In parallel with tissue expression, plasma and exosome fractions isolated from DOX-treated rats were found to be highly enriched in miR-34a with respect to control animals. The authors concluded that variation of miR-34a expression in the peripheral circulation could be potentially used as a marker of DOX-induced cardiac damage, although they did not provide evidence of a specific and restricted release of miR-34a from the heart.

## Circulating miRNAs

### miR-208a fails as circulating biomarker

Beside their mechanistic role in cardiac tissues, miRNAs started to be investigated also as circulating markers of drug-induced dysfunction. In 2015, Oliveira-Carvalho and colleagues tested the hypothesis that cardiac-specific miR-208a could be exploited as a biomarker of DOX-induced heart dysfunction in breast cancer patients [72]. For the very first time, they evaluated miR-208a expression levels in plasma of 59 female patients under the first round of chemotherapy. In particular, evaluation was conducted at the 3rd, 6th, 9th, and 12th week of treatment with DOX (60 mg/m^2^), cyclophosphamide (600 mg/m^2^), paclitaxel (80 mg/m^2^), or docetaxel (75 mg/m^2^). At the same time, cardiac damage was assessed by measuring serum cTnI and LVEF at echocardiography. Myocardial injury was considered present if cTnI serum levels were above a reference value of 40 pg/ml, while a significant decrease in LVEF with respect to baseline values indicated occurrence of cardiac dysfunction. Seven subjects (11.86% of patients) showed signs of cardiotoxicity, with increased cTnI and reduced LVEF vs. unaffected subjects. Unfortunately, no indication about timing and temporal extent of the abovementioned variations was reported, apart from troponin peak (12 weeks). Following assessment of heart damage, the authors evaluated the expression of miR-208a, but disappointingly, it was not detectable in any sample. Absence of inhibitors and technical issues was demonstrated by miR-1 detectability. The authors commented on these results by hinting that circulating miR-208a could increase only after acute heart injury, as shown in previously published studies [73, 74]. They concluded that miR-208a was probably not released into the bloodstream by chronically injured heart, therefore not representing a useful biomarker of DOX-induced cardiotoxicity in breast cancer patients.

### Perturbation of muscle-specific miRNAs upon cardiotoxic treatment

In 2015, starting from publicly available analyses of miRNA expression profiles in rat tissues, Nishimura and his group identified several miRNAs with very high expression in cardiac and skeletal muscle [74]. The idea at the base of the study was that highly expressed, tissue-specific miRNAs had a strong probability to represent circulating indicators of damage. More in detail, the authors investigated the possible use of a group of selected miRNAs as plasma biomarkers of cardiotoxicity in an animal model (male Sprague–Dawley rats), i.e., miR-208 (heart-specific); miR-1, miR-133a, and miR-133b (heart and skeletal muscle-specific); and miR-206 (skeletal muscle-specific). U6 snRNA was used as arbitrarily chosen internal control. To this end, they administered 4–5 rats/group with a single intravenous dose of either DOX (30 mg/kg) or isoproterenol (1 mg/kg), a known cardiac-damaging agent [75], or equivalent volumes of saline. Blood samples were collected after 2, 4, 8, and 24 h of treatment. Heart-specific miR-208 was significantly increased in plasma of isoproterenol-treated rats, but not in DOX-treated rats, as early as 2 h after administration. The other three heart/muscle miRNAs, miR-1, -133a, and -133b, showed upregulation both by isoproterenol and DOX treatment, although the former triggered a more marked response in terms of duration and extent of regulation. Interestingly, skeletal muscle-specific miR-206 increased only in DOX and not in isoproterenol-treated animals. These data, along with the observation that plasma cardiac troponin I (cTnI) and cTnT increased only in isoproterenol-treated rats but not in DOX rats (with opposite results for skeletal TnT plasma levels), clearly indicated that only isoproterenol triggered a detectable cardiac toxicity. Indeed, subsequent experiments in animals treated for 7 days with repeated dosing of isoproterenol showed histologic evidence of cardiac damage. The authors concluded that miR-208 should be considered as a promising plasma biomarker for cardiotoxicity in rats, as previously proposed by others [75]. This is the first work that casts doubts about the ability of DOX to cause cardiotoxicity, although this could be possibly due to an erroneous choice of evaluated cardiac damage makers. Indeed, the lack of histopathological findings in acute DOX treatment is not surprising, as it would be expected in a chronic setting.

### miR-1 is a possible circulating biomarker of DOX-induced cardiotoxicity

In a following study, Rigaud and coworkers took advantage of a branch of the CECCY trial (ClinicalTrials.gov NCT01724450), to investigate the suitability of a selected cluster of miRNAs as possible circulating markers of cardiotoxicity [76]. In particular, they enrolled 56 female patients undergoing chemotherapy with 4 cycles of DOX (cumulative dose of 240 mg/m^2^) and cyclophosphamide every 3 weeks, followed by paclitaxel for 12 weeks or docetaxel every 3 weeks. Three weeks after each cycle, they evaluated the expression of plasma miR-1, miR-133b, miR-146a, miR-208a, miR-208b, and miR-423-5p, together with cTnI. The choice of these miRNAs was based on previous literature regarding early circulating biomarkers of myocardial injury, HF, and/or drug-induced cardiotoxicity. Curiously, the cited literature referred only to heart tissue modulation of miR-208b [38] and to circulating levels of miR-1 and -133b in DOX-treated animals [74]. It must be noted that almost all of these miRNAs (but for miR-146a) were indeed previously proposed as circulating markers of cardiac diseases [17, 77], thus their choice was strongly supported by the literature. Conversely, the selection of miR-146a was probably dictated by its supposed regulation by DOX in vivo [35]. During treatment, cardiac function was assessed by echocardiography at baseline, at cycles 2 and 4 of DOX administration, and 9 and 12 months after the beginning of the chemotherapy. Cardiotoxicity was defined as a reduction in LVEF ≥ 10% with respect to baseline and/or a value of LVEF < 50% during or after chemotherapy. Mean plasma levels of cTnI increased after each cycle from baseline to the end of DOX treatment, although changes were not statistically significant, probably because of the great variability observed. Similarly, LVEF showed a modest, although not statistically significant, decreasing trend from baseline to cycle 4 in patients with cardiotoxicity. The difference between LVEF values in cardiotoxicity-affected vs. unaffected subjects widened even more at 9- and 12-month follow-up, but again the high variability observed in patients showing heart dysfunction hampered the reaching of statistical significance.

When analyzing miRNA expression, the authors once again confirmed that both miR-208a and miR-208b are not useful circulating markers, due to their undetectability. Conversely, miR-133b, -146a and -423-5p were detectable but did not show any appreciable variation of expression associated with cardiotoxicity onset. On the contrary, miR-1 showed an increasing trend over time in all subjects, in comparison to baseline levels, and a significant upregulation in cardiotoxicity-affected vs. non-cardiotoxic patients after cycle 2, 3, and 4. Moreover, miR-1 was the only circulating miRNA whose expression levels were associated with changes in LVEF, showing also a better area under the curve score than cTnI (0.851 vs. 0.544) in discriminating patients affected by cardiotoxicity from unaffected subjects.

An interesting finding of this work was that all detectable miRNAs showed an increasing trend during the treatment in all patients, albeit without statistical significance. A possible explanation might reside in the normalization strategy for miRNA expression levels, which was done using an exogenously added control miRNA, cel-miR-39. Indeed, this method does not take into account the possible variation of total RNA amount in each sample when fixed volumes of plasma are used. DOX treatment in the acute phase may increase oxidative stress and inflammation, which, in turn, could result in an increase of circulating RNA levels. Although this is a mere speculation driven by personal observations, the choice of endogenous miRNAs as references could have addressed this issue. In addition, as admitted by the authors, the choice of investigating a limited number of miRNAs greatly hindered the chances of identifying robust and specific markers of toxicity. Moreover, the complex dosing schedule of chemotherapy administration, the use of multiple drugs, and the limited follow-up and sample size strongly affected the results of this work. Anyway, it should be considered as the starting point for further studies on the same subject.

### Circulating miRNAs evaluation in young patients at risk of DOX-induced cardiotoxicity

Very recently, Leger and coworkers published the first investigation regarding plasma miRNAs as potential biomarkers of cardiotoxicity in children and young adults treated with anthracycline chemotherapy [78]. In their work, the authors used TaqMan Array Cards to screen 24 candidate miRNAs basing on their proposed role as circulating biomarkers of cardiovascular disease in 24 anthracycline-based and 9 non-cardiotoxic-agents-based chemotherapy cancer patients. The subjects were classified as belonging to different groups basing on troponin elevation. Out of the 24 AC patients, seven had acute troponin elevations (a post-AC cTnT concentration ≥ 5 ng/L compared to baseline), eight had chronic troponin elevations (a post-AC cTnT concentration ≤ 5 ng/L compared to baseline), and nine AC patients showed no cTnT increase at any time point. Three miRNAs, miR-1, miR-29b, and miR-499, were identified as presenting a significant upregulation in anthracycline (AC) patients (regardless of cTnT increase) vs. controls as early as 6 h after therapy administration (using miR-140 and miR-484 as normalizers). miR-1 and miR-499 remained increased up to 12 and 24 h. Interestingly, miR-1 and miR-29b expression seemed also to be correlated with the extent of anthracycline-induced injury measured by dosing high sensitivity cTnT. This work evidenced the potential of plasma miRNAs as early markers of anthracycline-mediated injury in the most relevant category of tumor patients, children, and young adults, since cardiotoxicity prevalence in long-term cancer survivors is known to be very high. As remarked by the authors, the heterogeneity and the low sample size of the investigated groups limited the clinical relevance of this work. At the same time, it represents the first step towards a new generation of biomarkers for the early identification of patients at high risk of anthracycline-induced cardiomyopathy.

## Summary and future directions

In the last few years, several groups took advantage of different animal models of DOX-induced cardiotoxicity in order to try to unravel the role of miRNAs in the harmful side effects of the drug. Differently from in vitro studies, the animal models present the clear advantage of a more close mimicry of the clinical setting. Unfortunately, a number of issues greatly limited the knowledge gained from these studies. The most common feature among the reviewed publications was the decision of electing to investigate only few, mostly “cardiac enriched” miRNAs (e.g., miR-208, miR-1, and miR-133). This choice obviously led to a strong limitation to the novelty and potential new findings. Moreover, a key issue was the less-than-optimal assessment of cardiotoxicity itself. Indeed, often the onset of heart dysfunction was not directly verified, either by instrumental evaluation (e.g., echography) or by dosage of circulating markers (e.g., troponins). Thus, the correlation between miRNA expression and cardiotoxicity was only assumed without verification. Another limitation was the lack of clear and detailed description of the methods used in many studies. Indeed, most authors generally referred to “heart samples” without specifying whether whole hearts or just parts of them were used in their analysis, thus making a comparison between different investigations difficult. In general, there is a clear need of increased consistency in the methods used. Finally, an issue of paramount importance regards the normalization strategy for a robust assessment of miRNA expression by RT-qPCR. This strongly affects the reproducibility of results and should be taken seriously into consideration for a possible exploitation of circulating miRNAs as biomarkers of DOX cardiotoxicity onset. Indeed, human-based studies pose greater challenges than animal models, because of the high variability in medical protocols and patients’ responses and the difficulties in patients’ recruitment and long medical follow-up. Thus, accurate and widely accepted research protocols should be implemented and adopted, to achieve reproducibility among different research groups and to pave the way to results of strong clinical relevance.
